# Positive breastfeeding experiences and facilitators in mothers of preterm and low birthweight infants: a meta-ethnographic review

**DOI:** 10.1186/s13006-021-00435-8

**Published:** 2021-11-27

**Authors:** Renée Flacking, Bente Silnes Tandberg, Hannakaisa Niela-Vilén, Rakel B. Jónsdóttir, Wibke Jonas, Uwe Ewald, Gill Thomson

**Affiliations:** 1 School of Health and Welfare, Dalarna University, Falun, Sweden; 2 Department of Paediatric and Adolescent Medicine, Drammen Hospital, Vestre Viken Hospital Trust, Drammen, Norway; 3 Lovisenberg Diaconal University College, Oslo, Norway; 4 Department of Nursing Science, University of Turku, Turku, Finland; 5 Faculty of Nursing, School of Health Sciences, University of Iceland, Reykjavik, Iceland; 6 Department of Women’s and Children’s Health, Karolinska Institutet, Stockholm, Sweden; 7 Department of Women’s and Children’s Health, Uppsala University, Uppsala, Sweden; 8 School of Community Health & Midwifery, Maternal and Infant Nutrition and Nurture (MAINN) research unit, University of Central Lancashire, Preston, UK

**Keywords:** Attunement, Breastfeeding, Breast milk, Feeding, Low-birthweight, Mother, Neonatal, NICU, Parent, Preterm

## Abstract

**Background:**

Most qualitative research on breastfeeding the preterm or low-birthweight (LBW) infant has focused on negative insights; there are no comprehensive insights into how, when and why mothers experience positive breastfeeding experiences. We aimed to address this knowledge gap by exploring what characterizes and facilitates a positive breastfeeding experience in mothers of preterm and/or LBW infants.

**Methods:**

A systematic review using meta-ethnographic methods was conducted. Search strategies involved a comprehensive search strategy on six bibliographic databases, citation tracking and reference checking. The analysis involved a reciprocal level of translation and a line of argument synthesis.

**Results:**

Searches identified 1774 hits and 17 articles from 14 studies were included, representing the views of 697 mothers. A positive breastfeeding experience was identified as being ‘attuned’. Three themes and eight sub-themes were developed to describe what characterizes attuned breastfeeding. ‘*Trusting the body and what it can do’,* concerned how attuned breastfeeding was facilitated through understanding the bodily responses and capacity and feeling comfortable with holding the infant and to breastfeed. ‘*Being emotionally present – in the here and now*’ described the importance of feeling relaxed and reassured. ‘*Experiencing mutual positive responses’,* illuminated how attunement was related to feelings of mutuality - when the mother recognises the infant’s cues, responds to these signals and receives a positive response from the infant. The key factors to facilitate attuned breastfeeding were opportunities for prolonged close physical contact with the infant, positive relationships with and support from staff and peers, and being facilitated to breastfeed when the infant showed feeding cues.

**Conclusions:**

This study provides new insights into what characterizes a positive breastfeeding experience and how staff can facilitate and enable mothers to achieve attuned breastfeeding. Improvements in units’ design, such as for rooming-in and having prolonged skin-to-skin contact, and care provided by knowledgeable, supportive and encouraging staff and peers, are crucial. The mother’s physical and emotional states and the infant’s behavioural responses and physiological signals should guide the process towards positive breastfeeding practices.

## Background

Over the last few decades, much attention has been paid to the intake of breast milk in preterm (< 37 gestational weeks) and low-birthweight infants (< 2500 g, LBW); breast milk provides nutritional, immunological, and neurological advantages compared to breast milk substitutes [1]. It has been suggested that even small changes in the prevalence of breast milk feeding may result in significant changes in health, healthcare costs, and economic productivity for preterm infants and their mothers [2]. Despite the overwhelming evidence of the value of breast milk for mothers and their preterm and/or LBW infants, there are wide variations in the initiation and duration rates of feeding breast milk [3–5], where preterm infants have shown to have a shorter breast milk feeding duration compared to term infants [6–8].

One of the major obstacles for breastfeeding (i.e., at breast) the preterm infant is the infant’s breastfeeding behaviour. Preterm infants’ ability to breastfeed is a maturational process and until the infant can be breastfed exclusively, mothers who want to breastfeed need to express their breast milk by pumping. In many settings, a range of non-evidence-based guidelines and care routines dictate that the infant should be of a certain gestational age when breastfeeding is initiated [9] or that the infant should tolerate full oral feeds before initiating breastfeeding [10]. Whereas a supportive neonatal unit context, including skin-to-skin contact, has been identified to facilitate the initiation and progression of breastfeeding for infants at lower gestational ages [11, 12]. Research has shown that infants maintain their physiological status when breastfed as early as 27 gestational weeks and can be exclusively breastfed at 32 weeks [13]. Thus, demonstrating that breastfeeding can be initiated despite an early gestational age. Scheduled feeding is also still mandated in policy in some units/countries, although there are indications that scheduled feeding could be replaced with more individualized and appropriate practices [14]. In the context of neonatal care, breastfeeding is often regarded as a productive process, with a primary focus on nutrition [15]. Such a focus fails to consider breastfeeding as being relational and valuable for emotional aspects such as comfort and pleasure, and can relegate breastfeeding into being an instrumental task based activity that is prone to problems and failure [16].

A few reviews have been conducted on parents’ experiences of breast milk feeding their preterm infants: parents’ experiences on factors that help or hinder breast milk supply [17]; factors that influence breastfeeding duration [18]; and mothers’ experiences with milk expression and breastfeeding [19]. Most qualitative research on breast milk feeding and breastfeeding the preterm infant has focused on negative insights, such as struggles with milk expression, conflicting advice from health professionals, lack of privacy or inadequate support and encouragement [20–23]. Currently there are no comprehensive insights into how, when and why mothers experience positive breastfeeding experiences. We aimed to address this knowledge gap by searching the literature to identify positive indicators and enablers for positive breastfeeding. We considered this approach to offer benefits to understand what a positive breastfeeding experience is, and how to facilitate this experience, emotionally and physically, for the mother and her infant. The aim of this meta-ethnographic review was therefore to explore what characterizes and facilitates a positive breastfeeding experience in mothers of preterm and/or LBW infants.

## Methods

### Design

We undertook a systematic review and used meta-ethnographic methods of Noblit and Hare to extract and analyse the findings [24]. A meta-ethnography is a commonly used method to combine and interpret findings from different qualitative methodological approaches [25]. During the process of this review, we adhered to the eMERGe Reporting Guidance, which was developed to ensure comprehensive and transparent reporting of meta-ethnographic research [25]. The review protocol was uploaded and published in PROSPERO [26].

### Search strategy

Search terms were identified using the PEO (Population; Exposure; Outcomes) structure. The terms were developed following scoping exercises and were agreed in collaboration with librarians at Karolinska Institutet, Sweden. An overview of the search string, according to the PEO structure, the inclusion and exclusion criteria and additional selection criteria (date of publication, study type and language) is presented in Table 1.

**Table 1:** Search terms and inclusion/exclusion criteria mapped to PEO framework

Criteria	Inclusion criteria	Exclusion criteria	Terms
Study population	Mothers of preterm (< 37 weeks gestation) or low birth weight (< 2500 g) infants who have been admitted to a neonatal unit Neonatal unit = Neonatal Intensive Care Unit (NICU), Special Care Baby Unit (SCBU)	Mothers whose infants were not admitted to neonatal unit, infants not preterm or low birth weight	mother* or maternal or women* low birth weight or preterm* or premature*
Exposure in context	Mothers’ experiences, perspectives, in neonatal units, in the transitional phase between hospital and home, or at home after discharge from neonatal unit		
Outcomes	Data concerns experiences of feeding infants their own breast milk	Never provided their own breast milk to their infant, experiences of expressing breast milk, experiences of providing bank milk	experience* or perception* or perspective* or view* breast feed* or breastfeed* or breast milk or breastmilk or breast pump* or breastpump* or human milk or lactation* or lactating
Date	2008 to present	Prior to 2008	limit to yr = “2008 -Current”
Study type	Qualitative studies, mixed-methods	Purely quantitative based studies, clinical case studies, reviews, theses, opinion pieces, grey literature.	ethnograph* or fieldwork or field work or focus group* or informant* or interview* or mixed method* or narration* or narrative* or open question* or participat* observation* or qualitative* or semi-structured or semistructured or thematic analys*
Language	English, Finnish, Swedish, and Norwegian published articles	Any other languages	limit to english or finnish or norwegian or swedish

Any study that described mothers’ experiences of breastfeeding their preterm (< 37 gestational weeks) or LBW (< 2500 g) infant were included. A broad definition of breastfeeding was used [27] where all methods of feeding the infant breast milk, such as the breast, bottle, cup, tube (gavage), were of interest. The experience of breastfeeding could relate to any time (from birth until weaning), and hence from neonatal units to at home after discharge. All qualitative studies were to be included, i.e., exploratory descriptive, narrative, case study, phenomenology, grounded theory, ethnography as well as mixed-methods studies that included sufficient qualitative data. The authors native language allowed for the inclusion of original studies published in different languages (i.e., English, Swedish, Norwegian, and Finnish). Only studies published from 2008 onwards were to be included. The rationale for this timeframe was based on the progression of family centred care and more neonatal units offering single-family rooms, systematic use of skin-to skin contact and early discharge, all practices that potentially affect breastfeeding in a positive way.

A comprehensive search strategy was used on six bibliographic databases: Medline (Ovid), Embase, Web of Science, PsycInfo (Ovid), CINAHL (Ebsco), and Global Index Medicus. Citation tracking and reference checking was also performed. Two librarians at Karolinska Institute University Library search consultation group undertook the database searches. All included papers from the searches were downloaded to an EndNote file and duplicates were removed.

### Study selection and appraisal

All abstracts were screened by at least two members of the review team against inclusion/exclusion criteria and papers were subsequently identified for full text review. All full text reviews were divided up across all members of the review team, and each paper was read in full by two reviewers. Agreements for inclusion were made by consensus, and any disagreements regarding inclusion were discussed with a third reviewer. The initial database searches were undertaken in October 2018, and again in June 2020.

All articles were quality appraised using the instrument developed by Walsh and Downe [28, 29]. The framework assesses studies against pre-defined criteria, and then allocates a score from A-D: A = no, or few flaws. The study credibility, transferability, dependability and confirmability are high; B = some flaws, unlikely to affect the credibility, transferability, dependability and/or confirmability of the study; C = some flaws that may affect the credibility, transferability, dependability and/or confirmability of the study; D = significant flaws that are very likely to affect the credibility, transferability, dependability and/or confirmability of the study. Only studies that scored C or higher were to be included in the final analysis.

Key data were extracted into a pre-designed template that included study aims/research question, methodology, sample size, participant characteristics, data collection methods, key findings/themes, and the quality appraisal rating (Table 2). Each paper was assigned to a lead reviewer (to extract the data) and a secondary reviewer (to check that all key issues had been recorded).

**Table 2:** Study characteristics and quality appraisal of included studies

Ref no.	Author Year	Aim	Country Type of unit(s)	Study design	Infants’ GA /weight at birth	Sample	Age of infant or timing when the study was conducted	Parent characteristics (age/parity)	Data collection methods	Data analysis methods	QA Grade
[30]	Bjork et al. 2012	To illuminate mothers experiences of breastfeeding a preterm infant in a neonatal ward	Sweden One 10-bed neonatal unit	Qualitative	27–36 gw	12 mothers who were breastfeeding at discharge	At home, 2–7 months post-discharge.	22–40 yrs., 7 had university education	Written text by mothers asked to write about their experiences of breastfeeding	Thematic analysis	B
[31]	Boucher et al. 2011	To explore the maternal experience of breastfeeding initiation and progression in the NICU	Canada One level III NICU	Qualitative descriptive	27–34 gw	10 mothers who had begun to breastfeed	At hospital, 2–7 weeks old	24–35 yrs., 7 had a secondary education, half of the mothers were primiparous	Face-to-face interviews	Qualitative content analysis	B
[32]	Breivold et al. 2019	To explore mothers’ experience after coming home from the hospital with a moderately to late preterm infant	Norway One unspecified neonatal unit	Qualitative explorative	30–35 gw	10 mothers	At home, 2–3 months after discharge	26–40 yrs., 8 Norweigan and 2 from Easetern Europe, 7 primiparous, 2 mothers with twins	Face-to-face interviews	Qualitative content analysis	A/B
[33]	Bujold et al. 2018	To explore whether mothers perceived expressing human milk for their infant in the NICU to be a closeness or separation experience and what factors gave rise to these perceptions	Canada One level III NICU	Qualitative descriptive	23–32 gw	15 mothers	At hospital, on average 37 days old at first data collection	26–44 yrs., 10 university education 10, primpiparous, 10 Canadian citizens	By the “Happy-app”, mother made voice recording where they described their experiences	Thematic content analysis.	A/B
[34]	Ericson et al. 2017	To explore mothers experiences of the proactive and reactive telephone support	Sweden Six NICUs	Qualitatively driven mixed-method evaluation	All < 37 gw with a mean GA of 34 gw	274 mothers provided written comments and 26 mothers were interviewed	At home, 8 weeks after discharge and at 6 and 12 months of infant age.	More than half had a university education, about 60% were primiparous, 6% not born in Sweden	Written comments to open-ended questions on questionnaires issued at 8 weeks after discharge and at 6 and 12 months of age. Telephone interviews at 8 weeks after discharge and at 6 months of age	Thematic network analysis	B
[35]	Ericson and Palmér 2019	To describe how mothers of preterm infants in Sweden experience breastfeeding support during the first 12 months after birth	Sweden Six NICUs	Hermeneutic approach	< 37 gw with a mean of 34 gw	151 mothers; 125 provided written comments, 12 interviewed, and 14 gave comments and interviewed	At home, 8 weeks after discharge and at 6 and 12 months of infant age.	Mean age was 30, 60% had a university education, 60% primiparous, 15% had twins, 6% not born in Sweden	Written comments to open-ended questions on questionnaires issued at 8 weeks after discharge and at 6 and 12 months of age. Telephone interviews at 8 weeks after discharge and at 6 months of age	Thematic network analysis	B
[36]	Flacking and Dykes 2013	To explore, in-depth, the impact of place and space on parents’ experiences and practices related to feeding their preterm babies in NICUs in Sweden and England	England and Sweden 2 NICUs in each country	Ethnographic	23–35 gw	52 mothers; 22 Swedish and 30 English	At hospital, observations were made throughout the hospital stay	19–45 years, 30 primiparous, 6 were not born in Sweden/England	Participant observations (210 h) and face-to-face interviews (96 h)	Grounded theory	A/B
[37]	Flacking and Dykes 2017	To explore perceptions and experiences of using a nipple shield among parents and staff in neonatal units in Sweden and England	England and Sweden 2 NICUs in each country	Ethnographic	Median 31 gw	12 mothers	At hospital, observations and interviews were made throughout the hospital stay	8 primiparous, 3 mothers with twins	Participant observations and face-to-face interviews	Thematic network analysis	B
[38]	Holdren et al. 2019	To understand how differences in neonatal care culture in two units in Finland and the US were translated to parents’ infant feeding experiences	Finland and the USA One level III NICU in each country	Unspecified qualitative	23–32 gw	15 mothers; 8 Finnish and 7 US mothers	In Finland: last week during the hospital stay, in the US: recently admitted to the NICU	20–44 years (mean 30), 6 mothers had twins	Interviews via telephone or face-to-face	Thematic narrative analysis	B/C
[39]	Ikonen et al. 2016	To describe maternal experiences of expressing breast milk for preterm or SGA infants.	Finland Internet-based	Descriptive	23–38 gw, mean of 31 gw	130 mothers	At home, 0–20 years (mean 4 years) of age	21–50 years (mean 34 years) 73% college or university degree, 23% twins or triplets, 58% previous breastfeeding experience	Open-ended questions in a web-survey	Qualitative inductive content analysis	B/C
[40]	Niela-Vilen et al. 2015	To describe the perceptions of breastfeeding mothers of preterm infants based on the postings in peer-support group discussions in social media.	Finland One level III NICU	Unspecified qualitative	Preterm infants	30 mothers of which 22 posted comments	At hospital and at home; 1st week post partum and then continuously during the first year	20–46 years (mean 29 years), 21 mothers were primiparous	Mothers posted comments on a secure FB page where only mothers who were recruited in a RCT could join. They accessed the FB group the 1st week postpartum and could continue to access the group at least until the infant turned 1 year	Inductive thematic analysis	C
[41]	Niela-Vilen et al. 2019	To describe maternal emotions regarding and insights into breastfeeding during the first year after a preterm birth.	Finland One level III NICU	Unspecified qualitative	25–35 gw	80 mothers	At infants’ discharge (hospital), and at 3, 6 and 12 months corrected age	21–46 years (median 31 years), 73% had a polytechnic/ university education, 70% primiparous, 11 mothers had twins	Answers on open-ended questions at discharge, 3 and 6 months. Telephone interviews or short questionnairs at 12 months.	Inductive thematic analysis	B/C
[42]	Palmér and Ericson 2019	To describe mothers’ experiences of breastfeeding their preterm infants from birth until 12 months after birth	Sweden Six NICUs	Unspecified qualitative	< 37 gw, median 34 gw	270 mothers	At home, 8 weeks after discharge and at 6 and 12 months of infant age.	Mothers had a mean age of 30 years, 51% had a university education, 59% primiparous and 32 mothers had twins	496 written comments to open-ended questions on questionnaires issued at 8 weeks after discharge and at 6 and 12 months of age.	Thematic network analysis	A
[43]	Parker et al. 2018	To examine the perceived barriers and facilitators of providing milk for very preterm infants during the hospitalization among Hispanic and non-Hispanic black mothers.	USA Two level III NICUs	Unspecified qualitative	24–37 gw median of 30 gw	23 mothers	At hospital and at home, when the infants were 2–18 months old	21–40 years, 12 Hipanic and 11 non-Hipsanic mothers, 2 mothers with twins	Interviews	Grounded theory approach	B
[44]	Radtke Demirci et al. 2015	To describe the process of breastfeeding establishment among late preterm mother-infant dyads.	USA One level III NICU	Constructivistic grounded theory	< 37 gw	10 mothers	At hospital 1–2 days after birth and then at home at 1, 2, and 6–8 weeks post partum	21–41 years, 7 had a college education, 5 primiparous, 2 mothers of twins,	Interviews with some mothers also contributing with e-mail or audioe diaries and video recordings with simulated recall interviewing	Grounded theory approach	B
[45]	Rossman et al. 2011	To describe the experiences of mothers with VLBW infants who received lactation care from certified Breastfeeding Peer Carers with special preparation for NICU care.	USA One level III NICU	Qualitative descriptive	24–31 gw and VLBW 511–1460 g	21 mothers	At hospital 12–80 days after birth during NICU stay	18–41 years, 17 had some college education, 10 primiparous	Interviews	Content analysis	A
[46]	Rossman et al. 2013	To describe the meaning of milk for mothers who were providing milk for their very low birth weight infants, hospitalised in the NICU	USA One level III NICU	Qualitative descriptive	23–33 gw and VLBW 600–1445 g	23 mothers	4–8 weeks of age	19–37 years, 5 had graduated from college education, 18 primiparous	Interviews and participant observations	Conventional (inductive) content analysis	A/B

Abbreviations: *GA* gestational age, *gw* gestational weeks, *NICU* Neonatal Intensive Care Unit, *VLBW* very low birth weight, *SGA* small for gestational age

### Strategy for data synthesis

An inductive and interpretative meta-ethnography approach was used. This approach distinguishes first, second and third order data [24, 25]. First order concerns participant quotes, second order the paper authors’ interpretations, and third order the review teams’ interpretations [24, 25]. Meta-ethnography involves identifying issues and concepts at the second order level, with this data then used by the review team to generate third order interpretations via mapping and organising the data into themes and associated sub themes; first order quotes were also extracted and used to authenticate and illuminate the interpretations. This process also involves translation whereby similarities (reciprocal) and contradicting or disconfirming (refutational) data are identified. Depending on the breadth of evidence identified, an overarching summary of all key issues (i.e., line of argument synthesis) is produced [24, 25]. In this review, we aimed to describe what characterized and facilitated positive breastfeeding experiences, rather than negative and/or contradictory experiences. Thus, our analysis focused on providing a reciprocal level of translation and a line of argument synthesis. The final themes and sub-themes were reviewed, refined and agreed by all authors.

## Results

**Fig. 1:**
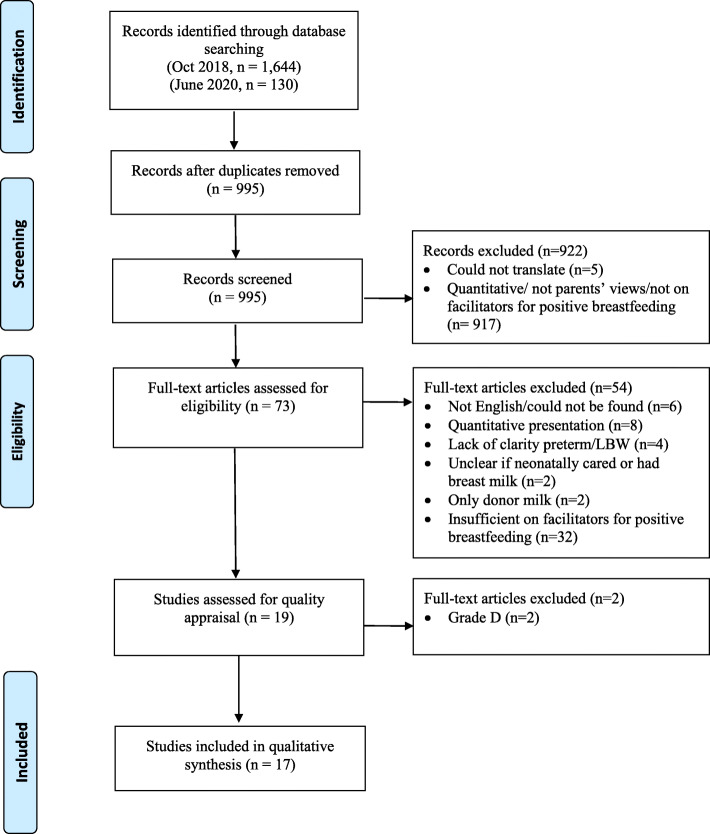
PRISMA flow diagram

In the original search, 1.644 hits were retrieved from the database searches and a further 130 during the updated search. No papers were identified via additional search methods. A total of 995 abstracts were screened against inclusion/exclusion criteria, 73 were reviewed as full-texts and 17 included in the final review (see PRISMA, Fig. 1).

The study characteristics and quality appraisal grade of the included studies are presented in Table 2. One of the studies was reported in three papers [34, 35, 42] and one study in two papers [36, 37], thus overall, the 17 articles comprised 14 studies. The 14 studies were conducted in the US (*n* = 4) [43–46], Finland (*n* = 3) [39–41], Sweden (*n* = 2) [30, 34, 35, 42], Canada (*n* = 2) [31, 33], Norway (*n* = 1) [32], Sweden and England (*n* = 1) [36, 37], and Finland and US (*n* = 1) [38]. Overall, these studies represent the views of 697 mothers. The focus for the papers were on breastfeeding experiences (*n* = 13) or the expression and provision of breast milk (*n* = 4). Ten papers focussed on experiences during the infant’s hospitalization, five on both the hospitalization and after discharge home, and two on experiences at home, after discharge. Although a broad definition of breastfeeding was used during the literature search and selection of papers, positive experiences of *feeding* the infant were described when the mother breastfed at breast. All studies included preterm infants, with some born as early as 23 weeks gestation. In all studies but one, the infant’s age ranged from 1 day to 18 months at the time of data collection. In the remaining study, the age of the infants ranged from newborn to 20 years of age [39]. Studies were conducted between 2008 and 2010 (*n* = 3), 2011–2015 (*n* = 6), and 2016–2019 (*n* = 3) and for two studies the year was not stated. The data collection methods generally involved interviews, but written texts in surveys, a single open-ended question, narratives in social media, or observations were also employed.

In the next section we first provide the line of argument synthesis to define a positive breastfeeding experience. We then detail the three themes and associated subthemes that describe the facilitators, situations and experiences that constitute and underpin positive attuned breastfeeding.

### A positive breastfeeding experience – feeling attuned

We defined a ‘positive breastfeeding experience’ as feeling attuned when breastfeeding. Feeling attuned was an experience that could occur briefly, or for a longer period, at any time from birth, regardless of breast milk intake, frequency and/or duration achieved. Mothers described attuned breastfeeding in terms of an emotional embodied connection [31, 38, 42], an experienced mutual interaction [36, 42], a “*symbiosis*” [41], or as a “*private moment of intimacy*” between them [41]. Attuned breastfeeding occurred when the mother trusted her body and what it could do, when the mother could be in the here and now and when she experienced mutual positive responses with her infant. Attuned breastfeeding was a joint reciprocal activity where both the infant and the mother contributed to the experience, and an interaction of psychological, social, physical and/or context related factors enabled the breastfeeding experience to be enjoyable and comfortable. Below we present the three themes and associated sub-themes that describe how attuned breastfeeding experience can be facilitated (see Table 3).

**Table 3:** Themes and subthemes linked to the individual papers

A positive breastfeeding experience – feeling attuned								
Themes	Trusting the body and what it can do			Being emotionally present – in the here and now		Experiencing mutual positive responses		
Subthemes	Trusting the body’s capacity	Transferring goodness	Trusting the “how to”	Feeling relaxed	Feeling reassured	Seeing and interpreting the infant’s cues	Responding to infant’s cues	Seeing a positive response from the infant
Bjork et al., 2012 [30]	x		x	x	x	x	x	x
Boucher et al., 2011 [31]	x	x	x			x		x
Breivold et al., 2019 [32]		x	x	x	x			
Bujold et al., 2018 [33]	x	x	x					
Ericson et al., 2017 [34]			x		x			
Ericson and Palmér, 2019 [35]	x		x		x			
Flacking and Dykes, 2013 [36]	x		x	x	x	x	x	x
Flacking and Dykes, 2017 [37]			x	x	x	x	x	
Holdren et al., 2019 [38]		x	x	x		x	x	x
Ikonen et al., 2016 [39]	x	x						
Niela-Vilen et al., 2015 [40]	x	x	x	x		x	x	x
Niela-Vilen et al., 2019 [41]		x	x				x	x
Palmér and Ericson, 2019 [42]	x	x	x	x	x		x	
Parker et al., 2018 [43]	x	x						
Radtke Demirci et al., 2015 [44]	x	x	x			x	x	x
Rossman et al., 2011 [45]	x	x			x			
Rossman et al., 2013 [46]	x	x			x			

### Trusting the body and what it can do

The theme of ‘trusting the body and what it can do’ comprises three subthemes, which describe how attuned breastfeeding is facilitated through understanding the bodily responses and capacity to produce breast milk (‘trusting the body’s capacity’), having faith and trust in the power of milk (‘transferring goodness’) and feeling comfortable with holding their infant and to breastfeed (‘trusting the ‘how to’).

#### Trusting the body’s capacity

Five papers highlighted how women’s trust in their bodies was important for a pleasurable experience of breastfeeding [30, 31, 36, 37, 42]. Boucher described this as “*the mother had to become more in tune with her body*” [31]. The studies described how mothers discovered their own physical limitations and gained a deeper understanding of how their mood, stress [31, 36, 39, 40, 43], and sleep [31, 33] impacted on their bodies ability to breastfeed. One mother in Boucher et al.’s study described:

“*To me the best thing to ensure effective breastfeeding is to know how your body works and what schedule works best, know your own system, your own schedule … and where’s your sleep at*.” [31].

Women’s trust in their bodies mainly related to their capacity to produce breast milk. This was reflected by a mother in Ikonen et al.’s study who stated: “*Beginning by expression was worth it. I was able to breastfeed when the time came for me to do it*.” [39]. In most studies the production of breast milk was seen as the initial start of a breastfeeding journey [31, 33, 35, 38–40, 42–44]. Mothers with very preterm infants described that it could take months until their infants had transitioned fully to breastfeeding and that this did not always occur during the infant’s hospitalization [32, 40]. The expression of breast milk was therefore an important “*stepping stone*” [33] and the beginning of a temporary process [30, 35] that would hopefully lead to the ultimate reward of breastfeeding [31, 33, 39, 42–44]. A mother in Bujold et al.’s study [33] described:

“*Pumping my milk, well it is part of the breastfeeding process. We’ve now started to feed him at the breast, it’s very motivating for me, it definitely makes me feel closer to my child. (P01)*” [33]

Strategies that helped milk production and women’s trust in their capacity to produce breast milk was holding the infant, having the infant skin-to-skin, or practicing breastfeeding [33, 40, 43]. A mother in Niela-Vilen et al.’s study [40] stated:

“*I believe that the daily kangarooing was really important because if the milk secretion didn’t start properly, but during kangarooing it started to flow. (025)*” [40]

Being able to express near the baby or in privacy by using drapes or screens [30, 33, 39] and having functional and easy to use equipment [33, 39, 43, 45] were regarded as facilitative. Staff or peer supporters encouragement, shared experiences, and support made mothers feel hope and security [30, 35, 43] in their bodies’ capacities and served as powerful motivators to initiate and sustain breast milk expression [33, 43, 45, 46].

#### Transferring goodness

A key facilitator for a positive breastfeeding experience was the belief or feeling that the provision of breast milk was a ‘transfer of goodness’. Mothers described that they had faith in the power of milk to increase their infant’s health, mitigate complications and help the infant grow [31, 32, 39, 41, 43, 44, 46]. Breast milk was described as a “*lifeline*” [39] and that providing milk was equivalent to “*giving life*” [46]. A mother in Rossman et al.’s study stated: “*I’m giving him life, medicine, food, and a part of me, all in a feeding every 2 hours.*” [46]. These beliefs mainly stemmed from the staff providing information and demonstrating positive attitudes towards breast milk and breastfeeding [40, 46], a positive breastfeeding culture, and the encouragement and support from the woman’s family or peers [33, 43, 45]. Mothers separated from their infants, initially or for most of the hospitalization, described that by expressing breast milk they had a purpose in being a mother and thereby felt connected [33, 38, 39, 41, 43, 46]. One mother stated: “*I think the breast milk—it’s me. I feel connected ‘cause my breast milk is a part of me. I mean, I’m makin’ this milk*.” [46]. Mothers often felt that producing breast milk was the only thing they could do and that providing breast milk was the only thing they could give [38, 39, 46]. For these women, expressing breast milk reminded them that they were a mother [41], and that they played a vital role in contributing to their infant’s care [33, 38, 39, 43].

#### Trusting the ‘how to’

An embodied feeling of trust in ‘how to’ breastfeed, including how to hold their infant and the techniques in breastfeeding, were reported in a number of studies [31, 35–38, 42, 44]:

“*It’s all about holding your breast; it’s really the techniques. You need to know [your breast]. And you have to feel comfortable with it*." [31]

Mothers needed to feel comfortable with holding their infant in order to experience a positive breastfeeding experience [31, 35, 36, 42, 44]. Some mothers described that they held their infant “*instinctively*” and felt comfortable with it [36], and while multiparous mothers found holding and breastfeeding easier [31], for others the feeling of being comfortable and secure progressed with time [31, 36].

In most studies, how to hold and how to breastfeed was practised in different degrees during the infant’s hospitalization. It was often a process that was balanced between the presence of staff providing support and advice, and mothers being able to do things in their own way; to “*stand on her own two feet*” [30]. For the support to be perceived as supportive, during the infants’ hospitalization, the transition to home or after discharge, staff needed to be attuned to the mother as an individual and provide support based on the mother’s needs and her infant’s needs [30, 32, 34, 35, 40]. Staff needed to be knowledgeable in breastfeeding preterm infants and provide information and practical guidance in a sensitive and timely manner; providing more and more pieces of information on e.g., different positions, how to manipulate the breasts or how to assess a proper latch [30–32, 34, 35, 37, 38, 40]. Mothers felt supported when staff were proactive, responsive (e.g., listened, showed interest), respectful (e.g., not judging or putting demands on the mother) [30–32, 34, 35], when they provided positive feedback and gave hope, and made mothers feel safe [30–32, 34]. A mother in Ericson et al.’s study stated:

*“If I had questions, they could answer them and they were very attuned. I thought the whole conversation was modelled after me. . . like the questions I had and what problems I had and so on. Then there was the encouragement. Sometimes you might not be so eager to continue breastfeeding after so many months of tube feeding and pumping to just get that encouragement, a little pat on the shoulder. (Interview 20)”* [34]

### Being emotionally present – in the here and now

In order to experience attuned breastfeeding, mothers need to feel emotionally present; that they can be in the here and now. Two subthemes describe the importance of physical closeness and privacy and the reassurance by staff and others for feeling emotionally present (‘feeling relaxed’ and ‘feeling reassured’).

#### Feeling relaxed

Mothers referred to how they needed to feel relaxed to achieve a state of attuned breastfeeding [30, 32, 36–38, 40, 42]. Feeling relaxed became easier by increased physical closeness and privacy [30], such as through having a single family room [36, 38]. In Flacking and Dykes study, one of the mothers stated: “*In there [the nursery], it’s a bit noisy and people are coming and going. She seems to have a better go if it’s quieter and I am relaxed*.” [36]. A private, familiar and safe space enabled mothers to act more freely and to immerse themselves in the breastfeeding experience [36, 38]. Holdren et al. described that by having a “*space to process their emotions and begin to get to know their infant*” [38 p. 6], mothers learnt to take care of themselves and their infant and become more autonomous:

*“It kinda felt like you can do . . . what you want more freely...because things like, like talking to the baby or singing to the baby . . . even though it kinda feels like the most normal thing, but when you have someone else in the room you kind of feel a bit more self conscious. And bursting into tears uhh next to someone who you don’t know . . . it’s not like the most, the most, most comfortable thing. (F2)”* [38]

For other mothers, the closeness and privacy did not occur until they came home to a familiar environment, “*a safe haven*” [32].

#### Feeling reassured

Another factor that enabled women to be in the here and now and to experience positive breastfeeding was the reassurance from staff and others that all was “*going well*”. Positive feedback from staff contributed to a feeling of security and calmness during hospitalization [30, 34, 35, 37] and at home [32, 34]. For some mothers, having the staff outside their private space, monitoring the infants, and entering their space when needed was reassuring [36]. A mother in Björk et al.’s study wrote:

*“It was very positive that the health professional was so calm and encouraging but not too pushy and stayed in the background. It is important that they step forward now and then and give advice but at the same time let me try on my own.”* [30]

This sense of reassurance was not only linked to breastfeeding per se but also to other aspects of being a mother of a preterm or LBW infant. Such reassurance could derive from staff [34, 35] but also from other parents of preterm infants who had “*walked in my shoes*” [45]. Thus, peer supporters through their “*mothering the mother”* [45] approach facilitated a state of calm and wellbeing [45, 46]:

“*They are really big on talking about postpartum depression and the counseling services available if I’m feeling stressed. So, they help with more than just breastfeeding. It’s kind of the whole package of dealing with having a baby in the NICU.* “ [45]

### Experiencing mutual positive responses

A sense of mutuality was a key facet of an attuned breastfeeding experience. Three subthemes describe this, where the mother recognise (‘*seeing and interpreting the infant’s cues’),* respond (‘*responding to infant’s cues’),* and then experience the infant’s response (‘*seeing a positive response from the infant’)*.

#### Seeing and interpreting the infant’s cues

Several studies described the importance of mothers being able to recognise their infant’s pre-feeding signals [31, 36–38, 40, 44] and their state of sleep-wakefulness [36, 37, 44]. Mothers described that being able to interpret their infant’s abilities, instincts and responses accurately made them feel that breastfeeding was a shared responsibility where they managed breastfeeding together as a team [30, 32, 38]. Boucher et al. described this as becoming “*an expert on observing and interpreting her infant’s behaviour*” [31]. One mother reported:

“*You’ve got to learn how to read your baby . . . know exactly how much milk she’s getting, learn if she’s eating effectively, if she’s latching properly. . . . I thought that everything with motherhood clicks instinctively. It doesn’t.*” [31]

Early positive feeding experiences, such as for mothers of infants with a low gestational age, related to ‘joyful’ and ‘fantastic’ experiences. Mothers were surprised by their infant’s competence and capacity the first time at the breast [30, 38], and the subtle, yet evident, cues their infants showed:

“*Well, of course it was really, well just fantastic . . . And even the first times that, well, not even breastfeeding, but when they said that you could like bring him next to your breast, and kind of like smell, and maybe lick a little bit. So that was for me kind of the experience. (F2)”* [38]

However, mothers also needed to have an awareness that the infant’s maturation to breastfeed would take time and that infants could not be rushed. This was described by a mother from Radtke-Demirci et al.’s study [44]:

*"[The connection between breastfeeding and bonding] is different since he’s not in that sleepy mode. Before I don’t even know if I saw it more as, like, nurturing. I’m just like, ‘This is just what I’m to do. He just needs to be held.’ He was supposed to still be inside of me, so of course I loved holding him then, but now I feel like it’s more of like a bonding . . .* ." [44].

The mothers understanding of the infant’s developing breastfeeding behaviour and interpreting signals were facilitated by staff describing infants’ cues and behaviour [30, 31, 34, 35, 38]. The studies that described this phenomenon referred to this as a ‘transfer of knowledge’, and how this reflected a staff member’s individual ‘trait’ rather than a joint unit responsibility. Some mothers also emphasized that continuity of care was needed to extend from hospital to soon after discharge via different forms of support e.g., proactive telephone support, domiciliary care provided by the neonatal unit and staff at other healthcare facilities [32, 34, 35].

#### Responding to infant’s cues

Mothers also needed to be able to respond to their infants’ pre-feeding signals or sleep-wakefulness cues [30, 31, 36, 38]. One mother in Flacking and Dykes (2013) study described her abilities and her twins’ cues:

“*They are lying with me so I know they get what they need. They relax more when they’re on me, in the sack* [kangaroo wrapping]*. But when they’re like this [in front of her] I can see them. Then it’s easier to see their signs of them being hungry. I try not to breastfeed less than every other hour. Sometimes they want to eat every hour. And sometimes, when I have put them down, they start to squirm and then I breastfeed again*,” [36].

Mothers’ abilities to respond to their infants’ signals was highly dependent on being physically present, having skin-to-skin contact, and being enabled or allowed to breastfeed when their infants’ signalled [30, 36, 38, 40], which created “*a window of opportunity for feeding in correspondence to the baby’s cues*” [36]. Mothers in Flacking and Dykes’s study [36] described how rooming-in meant that they did not miss their infant’s “*periods of awakeness*” [36]. One mother described how she and her partner became attuned to their infant when assigned a room of their own:

“*We withdrew from everything. We focused on him and it was peace and quiet and we could hear him. I saw that he was searching so I just put him at the breast and he started to suck and he hadn’t before. It was the breakthrough. There were just a few hours in between feedings. I was enabled freedom. I didn’t look at the clock but I did as he wanted. God how great! We were attuned to him. (MB6)”* [36]

Mothers described that when breastfeeding could be provided on an on-demand basis, breastfeeding was more enjoyable [30, 31, 44]. A mother in Flacking and Dykes’s study [37] described how staff wanted her to use a nipple shield in order for her infant to “*learn quicker*”. She refused as she felt that learning should happen on an individual basis, and that it was more important for her infant to “*suck and lick as he wants it*” [37].

With time, mothers’ and infants’ abilities to respond to each other was enhanced. A mother in Holdren et al.’s study stated: “*We kept practicing and we both improve [d] a lot. (F3)*” [38]. A mother in Niela-Vilen et al.’s study described her experiences of feeding her infant between the age of three to twelve months:

“*Breastfeeding is definitely one of the best things I have ever done. Both I and my baby enjoy and we will continue for a long time [at 3 months]. Breastfeeding is wonderful. The longer we breastfeed, the more pleasant it becomes. There is a special bond between me and my baby because of breastfeeding, and I wouldn’t change it for anything in the world [at 6 months]. It still is very comfortable, the baby enjoys as well [at 12 months]*” [41].

#### Seeing a positive response from the infant

The third facet of mutuality was the infant’s positive response. When this mutuality occurred, breastfeeding was perceived to be an enjoyable experience for both the mother and her infant [30, 31, 36, 37, 40–42, 44]. The interpretation of an infant’s response to breastfeeding depended on the mother’s previous experiences of breastfeeding, the mother’s knowledge about infants’ developing breastfeeding behaviours, and/or trust in her infant’s instincts [30, 40, 44]. A mother in Björk et al.’s study wrote:

"*With some arrangement with pillows and so on it started to work. My son started to look for the nipple and suck. Even though it was not for a long time I was at that moment thinking 'Yes he knows what to do'. And then the nervousness disappeared*." [30].

Mothers described a positive infant response during breastfeeding as being calm, alert, active, and that the infant sucked and swallowed [37, 40, 44] or fed “*efficiently*” [37, 42]. A more subtle experience was that infants enjoyed it [37, 38, 40, 41]. A mother in Holdren et al.’s study described: *“*. *.. and umm, and I feel like she was really enjoying it even though she didn’t get much out of it yet (F3)”* [38].

## Discussion

This systematic review and meta-ethnography aimed to define what characterizes and facilitates a positive breastfeeding experience in mothers of preterm and/or LBW infants. Insights into positive breastfeeding experiences were derived from 14 studies conducted in six countries. By using reciprocal translation, we identified characteristics of what constitutes a positive breastfeeding experience, an experience that we described as being ‘*attuned*’. Data was synthesized into three themes, ‘*trusting the body and what it can do’,* concerns how attuned breastfeeding is facilitated through understanding the bodily responses and capacity to produce breast milk, having faith and trust in the power of milk, and feeling comfortable with holding the infant and to breastfeed. The second theme, ‘*being emotionally present – in the here and now’* describes the importance of feeling relaxed and reassured through closeness, privacy, the support from staff, peers, and others for feeling emotionally present. The third theme, ‘*experiencing mutual positive responses’,* concerns how a feeling of mutuality arises when the mother recognises and interprets her infant’s cues, when she is enabled to respond to those signals and when she receives a positive response from her infant. In the following sections we first discuss attuned feeding drawing on key insights from the three themes, followed by a discussion on the three major facilitators for attuned feeding.

The phenomenon of attunement has been studied extensively, where the primary focus has been on behavioural, emotional and biological synchrony or mutuality between the mother and infant [47–50]. Our findings reflect those of Stern in terms of how attunement comprises, from the parental perspective, a sensitivity and responsiveness to infant cues and attentional states, and from the infant perspective the biological preparedness to engage in and also to anticipate attuned interactions, leading to exchanges of infant-parent mutually positive emotions [51].

The quality of parent-infant interaction and communication is an ongoing process that affects and contributes to the neurobiological regulation of the other [47, 52]. Breastfeeding is a key maternal activity, and the quality of this experience makes a substantial contribution to biological, social-emotional, and cognitive well-being [53, 54]. During embodied interactions such as breastfeeding, the mother and the infant need to coordinate their behaviours and bodies, both contributing to attunement. Embodied interactions in early life emerges from the dynamic interplay between signals arising inside the body and through affective exchanges [55]. As we identified within the review, trusting the body is crucial for attuned breastfeeding. Breastfeeding presents a situation of closeness and proximity, in which the mother feels attuned when she is emotionally present and in the here and now. When mothers feel relaxed during breastfeeding, the stress levels are reduced and infant behaviour during breastfeeding as well as in other behaviours such as sleep are positively affected [56]. But when mothers are preoccupied or have unresolved trauma they are less attuned to their infant’s cues during feeding, compared with those considered secure [57]. This was evident within the included papers in terms of how positive breastfeeding was associated with mothers who were emotionally present and felt connected.

Mother’s ability to see, interpret and respond to infant’s cues have mainly been explored in mothers of term infants. Biologically pre-programmed behaviours have been described by Widström and colleagues [58] and Matthiesen and colleagues [59] showing that when term infants are placed in skin-to-skin contact with their mothers immediately after birth, they interact both behaviourally and physiologically leading up to breastfeeding within one or two hours. Thus, this set of behaviours requires maternal attention, availability and physiological and psychological responsiveness. In the preterm dyad, more subtle movements and signs are present and therefore breastfeeding attunement requires a greater level of responsiveness to the infant’s cues and behaviours. Pre-feeding cues in preterm infants, just as in term infants, are not only cues of actual hunger but also an innate need to suck [60]. Breastfeeding can thus be the appropriate response to hunger but also to distress and pain [61] or to the infant’s need for increased pleasure [62]. Attuned breastfeeding may therefore be viewed as a comforting activity for the mother-infant dyad, in addition to the outcomes of feeding.

### Facilitators

Across the papers, three major facilitative factors were identified to enable attuned breastfeeding. First, ***being in closeness***; holding the infant or having the infant skin-to-skin enabled mothers to trust their bodies, become relaxed, and to see and respond to their infant’s signals. Montirossi and McGlone describe in their review [55], that the infant meets the mother’s body before (s) he meets the mother’s mind and the mother meets her infant’s body before the infant’s mind. Thus, the body comes first in the mother-infant interaction, which highlights the importance of physical proximity. Their review suggests that mother-infant interactions fluctuate between attuned and misattuned states, in which the latter can be repaired through e.g., skin-to-skin and affectionate touch and by a maternal sense of their internal bodily states. Skin-to-skin contact entails numerous physiological, physical, and psychological benefits for parents and infants, of which one is the evident positive short and long-term outcomes for breastfeeding [12].

The second major facilitator was the ***staff’s support and interpersonal relationship with the mother***. Schmied et al.’s findings from their meta-synthesis of women’s experiences of breastfeeding support [63], showed that mothers of term infants want staff to be ‘authentically present’ and have a ‘facilitative style’ in breastfeeding support. The findings from our study were similar. We found that mothers of preterm/LBW infants experienced good support when staff were attuned and responsive to mother’s and infant’s needs; reassuring, respectful, and encouraging; when they provided information and practical guidance in a sensitive and timely manner; and had a positive attitude towards breast milk and breastfeeding. Mothers from the included studies also described that staff needed to have specific knowledge in the breastfeeding behaviours of preterm infants and possess skills and knowledge in breastfeeding preterm terms [64]. As nurses in neonatal units provide support for breastfeeding during every shift [65], it is important to include all nurses in breastfeeding training programs to ameliorate the support provided to mothers and infants [66]. Unlike mothers of term infants, mothers of preterm/LBW infants have a much longer hospital stay with more staff encounters, which places a larger responsibility on staffs’ communication skills [67] and to ensure continuity and meaningful relationships [68]. Further, mothers need to be empowered by staff to be ‘in charge’ of breastfeeding and not become passive recipients of care and support [69]. Within settings where mothers are separated from their infants, it is hard for mothers to take active control to act freely and to gain trust in their capabilities [15]; this is contrary to units where the mother-infant dyad can spend unending time in close physical proximity [5]. However, regardless of the environmental features, staff support is crucial for breastfeeding preterm/LBW infants [70, 71].

The third major facilitator for attuned breastfeeding was ***being enabled to breastfeed*** when their infant signalled. By changing neonatal designs to include more private spaces and single-family rooms, the opportunities for mothers to support the infants’ resources and capacities (e.g., infants’ limited periods in alert behavioural state, strengths, muscle tone) unfolds. In line with the values from family centred care [72], an individual and dyadic approach to promote a more (neuro) developmental supportive breastfeeding context is needed. Cue-based feeding [73–78], responsive feeding [14, 79] and infant driven feeding [80–82], are all synonyms for a view that the infant’s signals (i.e., behavioural responses and physiological signals) should guide the process towards full breastfeeding or any oral feeding. However, these approaches to support the infant’s developing (breast) feeding behaviour have mainly been described as ‘suggestions’ in qualitative research or through quality improvement projects. Such approaches require more knowledge and support from the staff to guide mothers on their infant’s individual feeding development, and most of all a willingness to evaluate established practices, such as rigid scheduled feeding routines, that are potentially not conducive for mothers and infants.

### Strengths and limitations

The strengths of this review are the comprehensive search strategy, and a minimum of two reviewers being involved in each stage to enhance the rigour and trustworthiness. An evident limitation is that most studies were conducted in the Nordic countries. There are several potential reasons for this; the Nordic countries having a history of being pro-breastfeeding cultures and also that the progression towards family centred care has potentially moved faster than in other countries. Another limitation is that few papers have been published on positive experiences. Although a broad definition of breastfeeding was used during the literature search and selection of papers, positive experiences of feeding the infant were only described when the mother breastfed at breast in all papers. One reason for this could be that in many settings the norm and goal is to feed the infant directly at breast [5, 8]. When mothers do not achieve this goal but instead feed the infant by alternative means, i.e., a bottle, the overall experience becomes negative. Another reason could be that researchers regard ‘breastfeeding at breast’ as the focus of their paper and therefore not pay attention to positive experiences by other feeding methods. We would argue that attuned feeding occur irrespective of what method is used. Another limitation is the ‘staff-centeredness’ in many of the papers. A few of the papers described significant others’ (e.g., relatives, fathers, other mums in the neonatal units) attitudes towards breast milk and breastfeeding as being influential, but not in terms of actual support they provided. This could be due to the authoritative and institutionalised environment that neonatal units represent, in combination with a ‘medicalization’ of breastfeeding preterm infants, which reinforces staffs’ power and opportunities to assess, judge and evaluate the breastfeeding performance. As shown, no single study contributed to all the sub-themes, but the studies collectively enabled richer and more in-depth insights into what characterizes and facilitates a positive breastfeeding experience. Future research should focus on what constitutes attuned breastfeeding in different neonatal unit contexts, with different populations (e.g., from lower/middle income settings). Maybe more importantly, there is a need to evaluate different strategies and interventions in early breastfeeding, where the infants’ and mothers’ emotional and physical needs and capacities are acknowledged.

## Conclusions

This systematic review and meta-ethnographic methods identified positive breastfeeding experiences as being ‘attuned’. Attuned breastfeeding occurred when the mother trusted her body and what it could produce, when she could be emotionally and physical present in the here and now and when she experienced mutual positive responses with her infant. The most prominent facilitating factors for experiencing attuned breastfeeding were being in close physical proximity with the infant, meaningful and sensitive staff support and positive staff-mother interpersonal relationships, and being enabled to breastfeed when the infant signalled. This study offers new insights into how staff and gatekeepers in neonatal units can facilitate and enable mothers to achieve more positive breastfeeding experiences with a more prominent focus on the relational aspects of breastfeeding. Positive breastfeeding support requires a favourable environment which enhances mother-infant dyads closeness and trust in the mother’s and infant’s capacities.

## Data Availability

Data sharing is not applicable to this article as no datasets were generated or analysed during the current study.
